# Emergency department visits of newly diagnosed cardiovascular disease patients in Korea during the COVID-19 pandemic

**DOI:** 10.1038/s41598-023-50709-w

**Published:** 2024-01-03

**Authors:** Ji Yoon Baek, Seung Hee Seo, Sooyoung Cho, Jun-Bean Park, Bhumsuk Keam, Shin Hye Yoo, Aesun Shin

**Affiliations:** 1 Cancer Research Institute, Seoul National University, Seoul, South Korea; 2 Interdisciplinary Program in Cancer Biology Major, Seoul National University College of Medicine, Seoul, South Korea; 3 Integrated Major in Innovative Medical Science, Seoul National University Graduate School, Seoul, South Korea; 4 Medical Research Center, Genomic Medicine Institute, Seoul National University College of Medicine, Seoul, South Korea; 5 Division of Cardiology, Department of Internal Medicine, Seoul National University College of Medicine, Seoul, South Korea; 6 Cardiovascular Center and Department of Internal Medicine, Seoul National University Hospital, Seoul, South Korea; 7 Department of Internal Medicine, Seoul National University Hospital, Seoul, South Korea; 8 Center for Palliative Care and Clinical Ethics, Seoul National University Hospital, Seoul, South Korea; 9 Department of Preventive Medicine, Seoul National University College of Medicine, 103 Daehak-ro, Seoul, Jongno-gu 03080 Republic of Korea

**Keywords:** Cardiovascular diseases, Epidemiology, Health services, Public health

## Abstract

This study aimed to examine the impact of the COVID-19 pandemic on the emergency department (ED) visits of cardiovascular disease (CVD) patients. The customized data of the National Health Insurance Service (NHIS) from 2017 to 2020 were analyzed. CVD patients were defined by the code ‘V192’ based on the NHIS coverage benefit expansion policy. The number of ED visits of CVD patients, as well as executed procedures in 2020 (during the pandemic), were compared to the corresponding average numbers in 2018 and 2019 (prepandemic). Stratification by age group, residential area and hospital location was performed. The number of ED visits of newly diagnosed CVD patients decreased by 2.1% nationwide in 2020 (2018–2019: 97,041; 2020: 95,038) and decreased the most (by 14.1%) in March (2018–2019: 8539; 2020: 7334). However, the number of executed procedures increased by 1.1% nationwide in 2020 (2018–2019: 74,696; 2020: 75,520), while it decreased by 11.9% in April (2018–2019: 6603; 2020: 5819). The most notable decreases in the number of newly diagnosed CVD patients (31.7%) and procedures (29.2%) in March 2020 were observed in the Daegu·Gyeongbuk area. CVD patients living in the epicenter of the COVID-19 pandemic may experience difficulty accessing healthcare facilities and receiving proper treatment.

## Introduction

In March 2020, the World Health Organization (WHO) officially declared a global pandemic of COVID-19 since it quickly spread worldwide starting in Wuhan, China, in December 2019^1^. The first confirmed case of COVID-19 in Korea was reported on January 20, 2020^2^. To control the transmission of COVID-19, the Korean government and the Centers for Disease Control and Prevention (CDCP) implemented different levels of measures, such as social distancing or the redistribution of health care delivery systems. They provided guidelines to install exothermic detectors and to conduct simple health status questionnaires to screen suspected COVID-19 patients with fever or cough at the hospital entrance under social distancing circumstances^3–5^. Furthermore, they ordered the securing of designated hospitals or isolation beds for COVID-19 patients, and the hospitals started to triage COVID-19 patients. The government expanded the range of assignment of COVID-19 designated hospitals from 42 on February 21, 2020, to 69 on March 10, 2020^6^. The hospitals also rearranged areas to distinguish COVID-19 patients based on symptom assessment and triaged following the selective process protocol. For example, the hospitals assigned some emergency department (ED) units: a unit for screening patients and awaiting COVID-19 test results and an acute care unit divided into 3 degrees of severity (normal beds, negative-pressure emergency intensive care rooms, extremely intensive isolation rooms)^5^. The adjusted hospital systems restricted the accessibility of health care services for patients with other diseases who had not been infected with COVID-19, who were considered to experience collateral damage from the COVID-19 pandemic^7^. Collateral damage is also said to be the effect of distraction, including unintended disruption to health care delivery or collapse on the management of critical emergencies^8^.

Cardiovascular disease (CVD) patients have experienced collateral damage from the COVID-19 pandemic. Since most health care systems required these patients to be screened for COVID-19 and wait to be triaged or allocated a negative-pressure room for isolation, they could not access the health care system or receive proper care during the pandemic. An international survey conducted within 909 inpatient and outpatient centers from 108 countries showed a significant decline in CVD diagnostic tests or procedures, such as transthoracic echocardiography, by 42% from March 2019 to March 2020 and decreased further by 64% from March 2019 to April 2020^9^. A decrease in the number of CVD patients who admitted in EDs was observed in Saarland, Germany. The number of acute coronary syndrome (ACS) patients, including those with unstable angina pectoris, myocardial infarction with ST-elevation (STEMI), and myocardial infarction without ST-elevation (NSTEMI), decreased by 41% after the first COVID-19 case in calendar week 10 of 2020 compared to the same period in 2019 and decreased by 48% during the shutdown in weeks 12–16 of 2020^10^. These examples of collateral damage due to the COVID-19 pandemic still leave concerns regarding underdiagnosed harmful but treatable CVDs related to a reduction in ED visits.

In this study, we aimed to examine the impact of the COVID-19 pandemic on the emergency department visits of patients with a new diagnosis of CVD to address the potential collateral damage of the pandemic in Korea.

## Methods

### Ethics statement

The study was exempt from IRB review by the Institutional Review Board of Seoul National University College of Medicine/Seoul National University (IRB No. E-2010–002-1160), and written informed consent was waived because all analyses were performed by using secondary data for which personal information is unidentifiable.

### Study materials

A retrospective analysis of a cohort of patients from 2017 to 2020 was performed using the Customized Data of the National Health Information Database (NHID). The NHID is a public database based on the unidentifiable personal information of over 50 million people with the utilization of health care provided by the National Health Insurance Service (NHIS)^11^. The NHIS data include sociodemographic information, medical utilization data from clinics or pharmacies, death status, and the results of national health screening programs. The diagnosis of diseases and the types of medical services are presented as International Statistical Classification of Diseases and Related Health Problems 10th revision (ICD-10) codes^12^ and Korean Standard Classification of Diseases and Causes of Death (KCD-8) codes^13^.

### Study population

The eligible individuals were CVD patients who newly visited the ED and were diagnosed with the relevant diseases in 2018, 2019, and 2020. The CVD patients were defined by the code ‘V192’, granted if the patients had been charged for medical care benefits for CVDs and hospitalized to receive treatment for the diseases within 30 days based on the NHIS coverage benefit expansion policy. Each defined disease and procedure was based on the KCD-8 code as follows (Supplementary Table S1). CVD patients who newly visited the ED were those with no history of ED visits for the relevant diseases in the previous year. Of the total CVD patients who visited the ED from 2017 to 2020 (Supplementary Table S2), CVD patients who newly visited the ED between 2018 and 2020 were identified (Tables 1 and 2). The patients’ ED visits were identified by the code indicating ‘emergency department visit.’ The number of CVD patients who underwent the related procedures was also examined.

**Table 1:** Basic characteristics of the newly diagnosed cardiovascular disease patients.

	2018	2019	2020
Total, N	94,627	99,454	95,038
Sex, N (%)			
Male	63,185 (66.8)	66,905 (67.3)	64,942 (68.3)
Female	31,442 (33.2)	32,549 (32.7)	30,096 (31.7)
Age, N (%)			
0–39	4531 (4.8)	4436 (4.5)	4014 (4.2)
40–64	38,907 (41.1)	40,486 (40.7)	38,043 (40.0)
Over 65	51,189 (54.1)	54,532 (54.8)	52,981 (55.8)
Insurance type, N (%)			
Self-employed insured	28,195 (29.8)	30,698 (30.9)	29,645 (31.2)
Employee insured	60,142 (63.6)	62,335 (62.7)	59,303 (62.4)
Medical-aid beneficiary	6290 (6.6)	6241 (6.5)	6090 (6.4)
Income premium, N (%)			
Medical aid	6290 (6.6)	6241 (6.5)	6090 (6.4)
Bottom 25%	16,494 (17.4)	17,868 (18.0)	16,586 (17.5)
Bottom 50%	15,435 (16.3)	15,893 (16.0)	16,330 (17.2)
Top 50%	20,986 (22.2)	21,913 (22.0)	20,381 (21.4)
Top 25%	34,116 (36.1)	36,000 (36.2)	34,412 (36.2)
Missing/unknown	1306 (1.4)	1359 (1.4)	1239 (1.3)
Residential area, N (%)			
Capital^1^	42,365 (44.8)	44,898 (45.1)	43,009 (45.3)
Central^2^	14,130 (14.9)	14,668 (14.7)	13,548 (14.3)
Daegu·Gyeongbuk	10,338 (10.9)	10,760 (10.8)	10,163 (10.7)
Busan·Ulsan·Gyeongnam	15,603 (16.5)	16,305 (16.4)	15,436 (16.2)
Southwestern^3^	12,184 (12.9)	12,811 (12.9)	12,868 (13.5)
Missing/unknown	7 (0.0)	12 (0.0)	14 (0.0)
Location of medical institution, N (%)			
Capital^1^	49,831 (52.7)	52,889 (53.2)	49,996 (52.6)
Central^2^	11,161 (11.8)	11,440 (11.5)	10,538 (11.1)
Daegu·Gyeongbuk	9045 (9.6)	9430 (9.5)	9064 (9.5)
Busan·Ulsan·Gyeongnam	14,296 (15.1)	14,940 (15.0)	14,263 (15.0)
Southwestern^3^	10,294 (10.9)	10,755 (10.8)	11,177 (11.8)

^1^Capital (Seoul, Incheon, Gyeonggi).

^2^Central (Daejeon, Sejong, Gangwon, Chungbuk, Chungnam).

^3^Southwestern (Gwangju, Jeonbuk, Jeonnam, Jeju).

**Table 2:** The number and percentage difference of new cardiovascular disease patients who visited the ED by month, age and region.

		Jan	Feb	Mar	Apr	May	Jun	Jul	Aug	Sep	Oct	Nov	Dec	Sum
Total, nationwide	2018–2019	9187	7298	8539	8471	8201	7514	8367	7697	7009	8000	8287	8472	97,041
	2020	8601	7791	7334	7421	8135	8388	8553	7358	6865	8030	8441	8121	95,038
	Difference (%)	− 6.4	6.8	− 14.1	− 12.4	− 0.8	11.6	2.2	− 4.4	− 2.1	0.4	1.9	− 4.1	− 2.1
Age														
0–39	2018–2019	500	377	363	347	340	340	416	388	315	327	365	409	4,484
	2020	397	352	297	298	312	297	364	363	303	356	330	345	4,014
	Difference (%)	− 20.6	− 6.5	− 18.2	− 14.0	− 8.1	− 12.7	− 12.4	− 6.3	− 3.8	9.0	− 9.6	− 15.5	− 10.5
40–64	2018–2019	3712	2896	3426	3378	3309	3051	3479	3249	2965	3288	3424	3521	39,697
	2020	3385	3083	2988	2967	3169	3221	3425	3124	2796	3208	3401	3276	38,043
	Difference (%)	− 7.6	6.7	− 11.6	− 13.1	− 7.9	2.6	− 4.4	− 2.4	− 11.4	− 5.8	− 1.6	− 12.6	− 6.0
Over 65	2018–2019	4975	4026	4750	4747	4553	4123	4473	4061	3729	4386	4498	4543	52,861
	2020	4819	4356	4049	4156	4654	4870	4764	3871	3766	4466	4710	4500	52,981
	Difference (%)	− 3.1	4.2	− 16.0	− 14.6	− 1.8	13.4	1.2	− 7.2	− 5.5	0.2	3.2	− 5.0	− 2.8
Residential area														
Capital region^1^	2018–2019	4075	3227	3779	3828	3687	3398	3760	3463	3149	3582	3844	3842	43,632
	2020	3893	3504	3363	3371	3716	3703	3874	3261	3083	3663	3951	3627	43,009
	Difference (%)	− 4.5	8.6	− 11.0	− 11.9	0.8	9.0	3.0	− 5.8	− 2.1	2.3	2.8	− 5.5	− 1.4
Central region^2^	2018–2019	1417	1133	1300	1262	1222	1100	1250	1117	1014	1191	1188	1207	14,399
	2020	1270	1154	1103	1103	1111	1154	1175	1079	962	1111	1172	1154	13,548
	Difference (%)	− 10.4	1.9	− 15.1	− 12.6	− 9.1	4.9	− 6.0	− 3.4	− 5.1	− 6.7	− 1.3	− 4.4	− 5.9
Daegu·Gyeongbuk	2018–2019	1033	817	950	890	902	791	868	814	763	892	903	929	10,549
	2020	892	803	649	738	864	957	966	767	774	902	910	941	10,163
	Difference (%)	− 13.6	− 1.7	− 31.7	− 17.0	− 4.2	21.0	11.3	− 5.7	1.5	1.1	0.8	1.3	− 3.7
Busan·Ulsan·Gyeongnam	2018–2019	1460	1152	1415	1387	1365	1266	1432	1332	1,146	1311	1313	1379	15,954
	2020	1373	1323	1190	1196	1351	1374	1383	1217	1,100	1314	1330	1285	15,436
	Difference (%)	− 5.9	14.9	− 15.9	− 13.8	− 1.0	8.5	− 3.4	− 8.6	− 4.0	0.3	1.3	− 6.8	− 3.3
Southwestern region^3^	2018–2019	1204	970	1094	1104	1027	960	1057	971	937	1024	1039	1115	12,498
	2020	1173	1007	1028	1013	1092	1200	1153	1034	942	1037	1076	1113	12,868
	Difference (%)	− 2.5	3.9	− 6.0	− 8.2	6.4	25.1	9.1	6.5	0.6	1.3	3.6	− 0.1	3.0
Location of medical institution														
Capital region^1^	2018–2019	4844	3810	4471	4520	4320	4036	4441	4087	3680	4155	4482	4517	51,360
	2020	4561	4113	3888	3907	4265	4392	4553	3733	3567	4223	4554	4240	49,996
	Difference (%)	− 5.8	8.0	− 13.0	− 13.6	− 1.3	8.8	2.5	− 8.7	− 3.1	1.6	1.6	− 6.1	− 2.7
Central region^2^	2018–2019	1115	890	1014	984	971	879	961	861	785	970	931	944	11,301
	2020	980	909	859	851	895	867	878	866	761	868	916	888	10,538
	Difference (%)	− 12.1	2.2	− 15.2	− 13.5	− 7.8	− 1.3	− 8.6	0.6	− 3.1	− 10.5	− 1.6	− 5.9	− 6.8
Daegu Gyeongbuk	2018–2019	911	717	812	778	808	663	765	707	679	797	779	825	9238
	2020	817	696	555	646	778	813	852	694	694	819	857	843	9064
	Difference (%)	− 10.3	− 2.9	− 31.7	− 16.9	− 3.7	22.6	11.4	− 1.8	2.3	2.8	10.1	2.2	− 1.9
Busan·Ulsan·Gyeongnam	2018–2019	1330	1061	1311	1285	1252	1149	1307	1212	1048	1207	1202	1257	14,618
	2020	1251	1208	1116	1124	1253	1269	1293	1140	1007	1214	1216	1172	14,263
	Difference (%)	− 5.9	13.9	− 14.8	− 12.5	0.1	10.4	− 1.1	− 5.9	− 3.9	0.6	1.2	− 6.7	− 2.4
Southwestern region^3^	2018–2019	988	822	932	906	851	788	894	832	818	872	894	930	10,525
	2020	992	865	916	893	944	1,047	977	925	836	906	898	978	11,177
	Difference (%)	0.5	5.3	− 1.7	− 1.4	10.9	32.9	9.3	11.2	2.3	3.9	0.5	5.2	6.2
Concordance of residential area and location of medical institution														
Capital Region^1^	2018–2019	3968	3130	3678	3712	3575	3296	3655	3360	3061	3466	3741	3724	42,363
	2020	3787	3413	3285	3292	3609	3605	3792	3156	2985	3548	3834	3534	41,840
	Difference (%)	− 4.6	9.1	− 10.7	− 11.3	1.0	9.4	3.8	− 6.1	− 2.5	2.4	2.5	− 5.1	− 1.2
Central region^2^	2018–2019	998	804	912	876	862	774	866	769	703	855	833	848	10,098
	2020	887	819	782	776	799	777	795	768	671	772	822	797	9465
	Difference (%)	− 11.1	1.9	− 14.3	− 11.4	− 7.3	0.4	− 8.2	− 0.1	− 4.6	− 9.7	− 1.3	− 6.0	− 6.3
Daegu·Gyeongbuk	2018–2019	857	666	761	729	753	625	702	659	634	744	734	773	8636
	2020	753	662	540	621	720	770	811	643	648	763	793	798	8522
	Difference (%)	− 12.1	− 1.0	− 29.0	− 14.8	− 4.4	23.2	15.5	− 2.4	2.2	2.6	8.1	3.2	− 1.3
Busan·Ulsan·Gyeongnam	2018–2019	1281	1006	1242	1225	1197	1097	1250	1159	1000	1157	1147	1207	13,966
	2020	1197	1160	1074	1081	1195	1204	1245	1079	963	1162	1165	1127	13,652
	Difference (%)	− 6.5	15.3	− 13.5	− 11.8	− 0.2	9.8	− 0.4	− 6.9	− 3.7	0.4	1.6	− 6.6	− 2.2
Southwestern region^3^	2018–2019	957	788	890	867	812	748	855	785	783	830	850	881	10,045
	2020	938	829	876	851	902	1000	941	873	794	857	862	925	10,648
	Difference (%)	− 2.0	5.2	− 1.5	− 1.9	11.2	33.7	10.1	11.2	1.4	3.3	1.4	5.0	6.0

^1^Capital (Seoul, Incheon, Gyeonggi).

^2^Central (Daejeon, Sejong, Gangwon, Chungbuk, Chungnam).

^3^Southwestern (Gwangju, Jeonbuk, Jeonnam, Jeju).

### Analyses

The age group was divided into 3 categories: under 40, from 40 to 64, and older than 65 years. The insurance type was divided into 3 types: self-employed insured, employee-insured, and medical-aid beneficiary. The insurance premium was categorized into 5 groups: medical aid, the bottom 25%, the bottom 50%, the top 50%, and the top 25%. The regions were categorized into 5 categories: the Capital (Seoul, Incheon, Gyeonggi), Central (Daejeon, Sejong, Gangwon, Chungbuk, Chungnam), North of the Southeastern (Daegu, Gyeongbuk), South of the Southeastern (Busan, Ulsan, Gyeongnam) and Southwestern (Gwangju, Jeonbuk, Jeonnam, Jeju) regions.

The reference period was from 2018 to 2019, before the COVID-19 pandemic. The pattern of the number of daily newly confirmed COVID-19 cases in Korea in 2020 was observed by using data from the Korea Disease Control and Prevention Agency (KDCA)^14^. The average number of patients who newly visited the ED and those who underwent the related procedures from 2018 and 2019 for each month were compared to the corresponding number in 2020. The percentage difference was also obtained by dividing the difference in the number between two periods. Stratified analyses by age group, residential area, and location of the medical institution were conducted. In addition, the concordance between residential areas and the location of the hospital visited by the CVD patients was calculated to reflect the effect of proper response on emergent CVD patients.

All analyses were conducted using Microsoft Excel (Microsoft, Redmond, WA, USA) and SAS (SAS Institute Inc., Cary, NC, USA).

## Results

### Pattern of the COVID-19 pandemic in 2020

**Figure 1:**
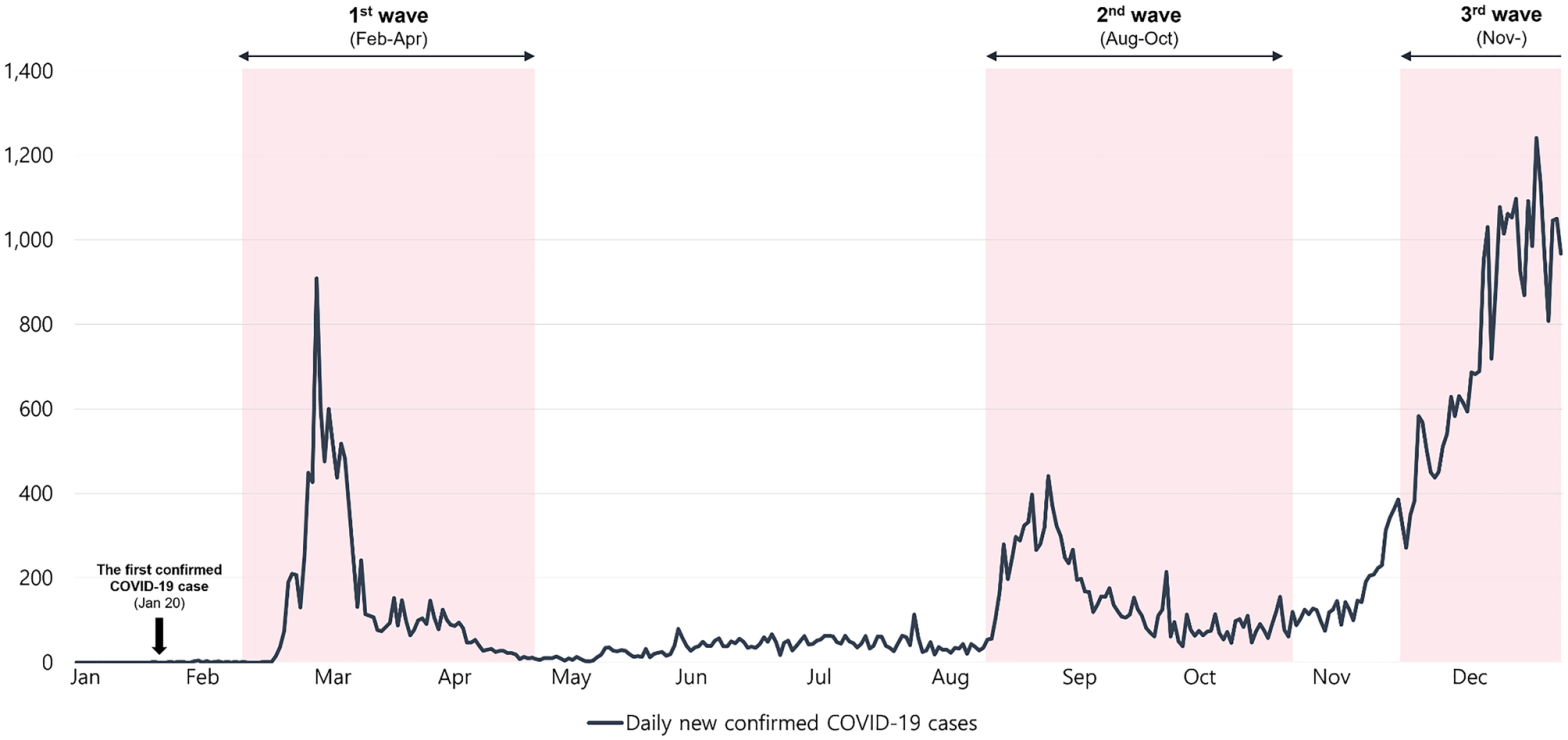
The number of daily newly confirmed COVID-19 cases in Korea in 2020.

In 2020, there were three waves of COVID-19 in Korea (Fig. 1). The first COVID-19 epidemic wave struck the Daegu·Gyeongbuk region in March 2020, leading to the introduction of the first intensive social distancing measures nationwide in April^2^. The second wave was centered in the capital region in August 2020^2^. The third wave started in the capital and central regions and spread nationwide in December 2020^2^.

### Comparison of basic characteristics

We compared the basic characteristics of the CVD patients, including sex, age, insurance type, insurance premium, residential areas, and the location of the medical institution, from 2018 to 2020. The total number of CVD patients who visited the ED during the study period was 108,841 in 2018, 114,579 in 2019 and 111,500 in 2020 (Supplementary Table S2). Among these patients, 94,627 newly visited the ED in 2018, 99,454 newly visited in 2019 and 95,038 newly visited in 2020 (Table 1). The proportions of sex, age, insurance type, income level, residential area, and location of the medical institution remained similar over the 3 years. During the observed years, the proportion of males (66.8–68.3%) and those aged older than 65 years (54.1–55.8%) was higher than their counterparts.

### The number of visits to emergency department

The total number of CVD patients who were newly diagnosed and visited the ED decreased by 2.1% nationwide in 2020 compared to the corresponding average numbers in 2018 and 2019 (2018–9: 97,041; 2020: 95,038) (Table 2). The decrease was observed to be the most significant at 14.1% nationwide in March 2020 (2018–9: 8539; 2020: 7334), while it increased the most by 11.6% in June 2020 (2018–9: 7514; 2020: 8388). Comparing the stratified age groups, the group aged older than 65 years showed the sharpest decrease in March 2020 by 16.0% (2018–9: 4750; 2020: 4049), and the group aged 40 to 64 years had the sharpest decrease by 13.1% in April 2020 (2018–9: 3378; 2020: 2967), followed by that of 11.6% in March 2020.

**Figure 2:**
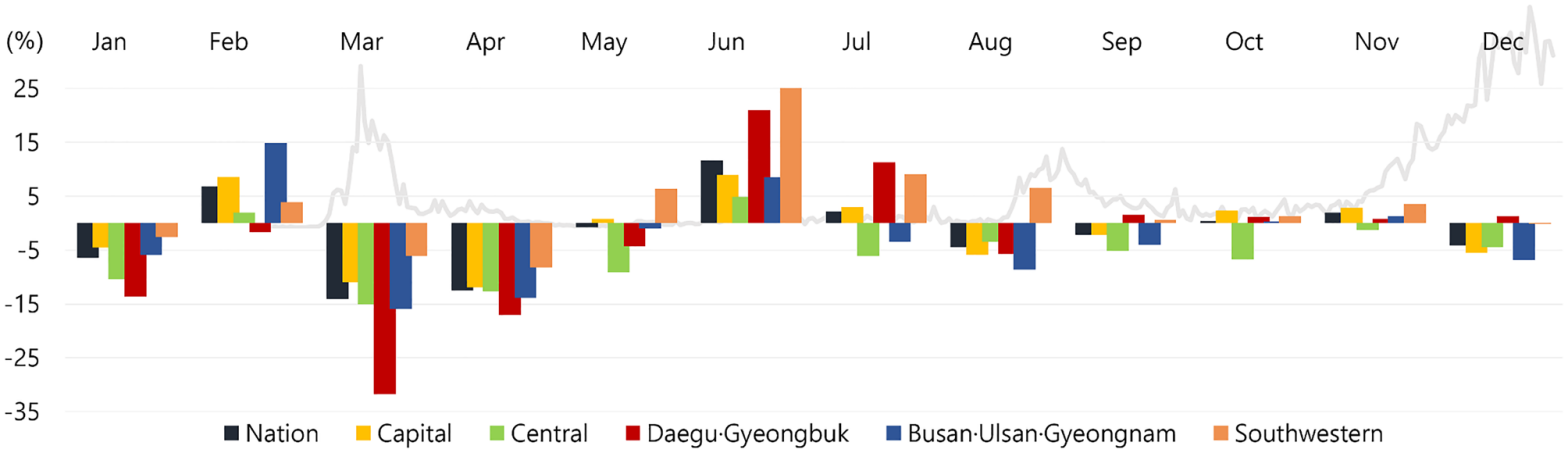
The percentage difference in the number of new ED visits of CVD patients by residential area in 2020 compared to 2018–2019. *ED* Emergency department, *CVD* Cardiovascular disease.

The total number of new CVD patients with ED visits decreased in all regions except for the southwestern region (Table 2). The sharpest decline of 31.6% was observed in the Daegu·Gyeongbuk area in March 2020 (2018–9: 950; 2020: 649) and in the capital region in April 2020 by 11.9% (2018–9: 3828; 2020: 3371) (Fig. 2). Similar patterns were observed regarding the location of the medical institutions, with a decline by 31.7% in the Daegu·Gyeongbuk area in March 2020 (2018–9: 812; 2020: 555) and 13.6% in the capital region in April 2020 (2018–9: 4520; 2020: 3907). The concordance between the residential area and the location of the medical institutions decreased in all regions except for the southwestern region (6.0%), with the sharpest decrease by 6.3% in the central region. Likewise, the Daegu·Gyeongbuk area showed a 29.0% decrease in March 2020 (2018–9: 761; 2020: 540) and a decrease of 11.3% in the capital region in April 2020 (2018–9: 3712; 2020: 3292). The differences in the number of new CVD patients with ED visits based on the other characteristics, including sex, insurance type and income level, are shown in Supplementary Table S3.

### The number of emergency department visits and undergone procedures

While the total number of CVD patients with new diagnoses and ED visits decreased nationwide in 2020, that of those who had undergone procedures showed an increase of 1.1% in 2020 (2018–9: 74,696; 2020: 75,520) (Table 3). In April 2020, the sharpest decline of 11.9% was observed. Among the age groups, the number of individuals aged older than 65 years decreased the most by 12.1% in April 2020, as did the number of individuals aged between 40 and 64 years (12.2%). The capital region and the southwestern region showed an increase in the total number; however, that of the central region and Daegu·Gyeongbuk decreased. The most significant reduction was observed in Daegu·Gyeongbuk in March 2020. The changes based on the other characteristics, including sex, insurance type and income level, are shown in Supplementary Table S4.

**Table 3:** The number and percentage difference of new cardiovascular disease patients who visited the ED and underwent procedures by month, age and region.

		Jan	Feb	Mar	Apr	May	Jun	Jul	Aug	Sep	Oct	Nov	Dec	Sum
Treated	2018–2019	6958	5536	6471	6603	6328	5787	6544	6018	5322	6193	6491	6447	74,696
	2020	6811	6228	5841	5819	6497	6741	6904	5763	5473	6299	6721	6405	75,502
	Difference (%)	− 2.1	12.5	− 9.7	− 11.9	2.7	16.5	5.5	− 4.2	2.9	1.7	3.5	− 0.7	1.1
Age														
0–39	2018–2019	311	245	218	225	219	212	273	282	196	211	238	261	2,887
	2020	270	234	206	214	221	212	244	246	210	235	224	232	2,748
	Difference (%)	− 13.0	− 4.5	− 5.3	− 4.9	1.1	0.00	− 10.5	− 12.6	7.4	11.6	− 5.7	− 10.9	− 4.8
40–64	2018–2019	2803	2182	2600	2612	2538	2353	2708	2517	2230	2518	2677	2691	30,428
	2020	2662	2454	2355	2294	2486	2570	2778	2430	2233	2496	2692	2593	30,043
	Difference (%)	− 5.0	12.5	− 9.4	− 12.2	− 2.03	9.2	2.6	− 3.5	0.1	− 0.9	0.6	− 3.6	− 1.3
Over 65	2018–2019	3844	3109	3654	3767	3572	3222	3564	3219	2896	3465	3577	3496	41,382
	2020	3879	3540	3280	3311	3790	3959	3882	3087	3030	3568	3805	3580	42,711
	Difference (%)	0.9	13.9	− 10.2	− 12.1	6.1	22.9	8.9	− 4.1	4.6	3.0	6.4	2.4	3.2
Residential area														
Capital region^1^	2018–2019	3121	2444	2838	2995	2850	2633	2975	2721	2412	2781	3042	2913	33,721
	2020	3179	2808	2709	2692	2987	3026	3186	2637	2510	2903	3215	2916	34,768
	Difference (%)	1.9	14.9	− 4.6	− 10.1	4.8	15.0	7.1	− 3.1	4.1	4.4	5.7	0.1	3.1
Central region^2^	2018–2019	1072	862	991	975	952	840	966	874	765	917	917	926	11,056
	2020	1001	909	884	838	900	913	917	815	764	881	901	924	10,647
	Difference (%)	− 6.6	5.5	− 10.8	− 14.1	− 5.5	8.7	− 5.0	− 6.8	− 0.1	− 3.9	− 1.7	− 0.2	− 3.7
Daegu·Gyeongbuk	2018–2019	791	630	719	697	690	613	680	631	578	692	704	710	8132
	2020	674	638	509	585	685	748	781	587	619	691	728	727	7972
	Difference (%)	− 14.8	1.4	− 29.2	− 16.1	− 0.7	22.0	14.9	− 6.9	7.1	− 0.1	3.5	2.5	− 2.0
Busan·Ulsan·Gyeongnam	2018–2019	1094	889	1090	1074	1057	981	1120	1042	875	1023	1023	1045	12,310
	2020	1083	1083	960	945	1112	1121	1104	927	866	1028	1060	1016	12,305
	Difference (%)	− 1.0	21.8	− 11.9	− 12.0	5.2	14.3	− 1.4	− 11.0	− 1.0	0.5	3.6	− 2.8	− 0.0
Southwestern region^3^	2018–2019	881	712	833	862	779	720	805	750	691	780	806	854	9471
	2020	874	790	778	759	813	933	914	797	711	793	816	822	9800
	Difference (%)	− 0.7	11.0	− 6.6	− 12.0	4.4	29.6	13.5	6.3	2.9	1.7	1.3	− 3.7	3.5
Location of medical institution														
Capital region^1^	2018–2019	3634	2825	3291	3449	3281	3052	3433	3166	2786	3163	3471	3359	38,908
	2020	3662	3259	3089	3069	3406	3518	3659	2974	2851	3296	3673	3365	39,821
	Difference (%)	0.8	15.4	− 6.1	− 11.0	3.8	15.3	6.6	− 6.4	2.3	4.2	5.8	0.2	2.4
Central region^2^	2018–2019	860	700	797	795	780	690	756	684	608	762	747	747	8924
	2020	783	733	700	660	727	691	713	656	611	703	698	726	8401
	Difference (%)	− 8.9	4.7	− 12.2	− 16.9	− 6.8	0.2	− 5.6	− 4.1	0.5	− 7.7	− 6.6	− 2.8	− 5.9
Daegu·Gyeongbuk	2018–2019	709	568	633	622	631	521	612	557	519	634	619	649	7272
	2020	617	559	449	514	617	653	693	533	561	635	693	653	7177
	Difference (%)	− 12.9	− 1.6	− 29.1	− 17.3	− 2.2	25.3	13.3	− 4.3	8.1	0.2	12.0	0.6	− 1.3
Busan·Ulsan·Gyeongnam	2018–2019	1019	830	1025	1017	984	909	1035	953	801	956	955	972	11,453
	2020	995	1003	908	903	1039	1054	1045	879	813	964	975	938	11,516
	Difference (%)	− 2.3	20.8	− 11.4	− 11.2	5.6	16.0	1.0	− 7.7	1.5	0.9	2.1	− 3.5	0.6
Southwestern region^3^	2018–2019	737	613	726	722	652	615	710	659	608	679	700	721	8140
	2020	754	674	695	673	708	825	794	721	637	701	682	723	8587
	Difference (%)	2.3	10.0	− 4.3	− 6.8	8.6	34.2	11.9	9.5	4.9	3.2	− 2.5	0.3	5.5
Concordance of residential area and location of medical institution														
Capital region^1^	2018–2019	3055	2381	2769	2905	2771	2561	2898	2644	2351	2694	2963	2826	32,815
	2020	3105	2752	2659	2637	2916	2951	3123	2566	2442	2818	3138	2852	33,959
	Difference (%)	1.7	15.6	− 4.0	− 9.2	5.2	15.2	7.8	− 3.0	3.9	4.6	5.9	0.9	3.5
Central region^2^	2018–2019	782	637	729	710	700	613	684	619	549	675	670	674	8040
	2020	714	671	649	606	660	627	650	587	545	633	631	659	7632
	Difference (%)	− 8.7	5.4	− 11.0	− 14.7	− 5.7	2.4	− 5.0	− 5.2	− 0.6	− 6.2	− 5.8	− 2.2	− 5.1
Daegu·Gyeongbuk	2018–2019	672	527	596	588	591	492	565	519	484	595	585	607	6817
	2020	572	533	437	499	572	625	661	500	528	597	642	620	6786
	Difference (%)	− 14.8	1.2	− 26.7	− 15.1	− 3.1	27.2	17.1	− 3.6	9.1	0.4	9.7	2.1	− 0.5
Busan·Ulsan·Gyeongnam	2018–2019	981	790	975	973	943	869	993	913	766	920	913	932	10,965
	2020	953	966	873	867	993	998	1007	834	774	926	939	906	11,036
	Difference (%)	− 2.9	22.3	− 10.4	− 10.9	5.4	14.9	1.4	− 8.6	1.0	0.7	2.9	− 2.8	0.7
Southwestern region^3^	2018–2019	717	590	694	696	626	587	680	625	582	651	670	688	7804
	2020	716	651	668	647	678	789	765	685	607	668	656	686	8216
	Difference (%)	− 0.1	10.3	− 3.8	− 7.0	8.4	34.4	12.5	9.7	4.3	2.7	− 2.0	− 0.3	5.3

^1^Capital (Seoul, Incheon, Gyeonggi).

^2^Central (Daejeon, Sejong, Gangwon, Chungbuk, Chungnam).

^3^Southwestern (Gwangju, Jeonbuk, Jeonnam, Jeju).

## Discussion

The total number of CVD patients with new diagnoses and ED visits decreased. In particular, the Daegu·Gyeongbuk area experienced the most significant decline in March 2020. This indicates that the Daegu·Gyeongbuk region experienced the most severe collateral damage from the COVID-19 pandemic.

The onset of the early COVID-19 epidemic in Korea appeared as a pattern of sporadic clusters. The Daegu·Gyeongbuk region was confronted with the crisis of the first epidemic wave caused by a religious group, Shincheonji, in the middle of February 2020. With the soaring number of confirmed cases in the Daegu·Gyeongbuk region in March 2020, intensive social distancing was applied nationwide until the beginning of May. Then, the second wave appeared similar to the first with religious activities and rallies in the capital region in August. The third wave started in December with spreading nationwide through the various clusters of hospitals and religious facilities, and even family-to-family infections occurred^2^. Our findings demonstrate that dramatic declines during the pandemic were observed in accordance with the epidemic periods, when the first and second epidemic waves struck, followed by a surge in COVID-19 cases nationwide. It also emphasizes that the regions with COVID-19 clusters were affected more severely; for example, there were immense pressures on the health care delivery system in Daegu after 121 health care workers were infected with the COVID-19 virus^5^.

Surveys conducted in the United States demonstrated that older people tended to be more compliant with social distancing regulations, and younger people had greater concern about COVID-19 infection or being quarantined^15,16^. This can explain the results of our study, which showed a relatively low decline in ED visits among cardiovascular patients older than 65 years, while those among patients under 40 years old decreased by more than 10%. Given the small number of young age groups in our study, it was reported from a multicenter study with five different major children’s hospitals in Korea that the number of pediatric ED visits during the COVID-19 pandemic was 58.1% lower than that in the previous years of 2018 and 2019^17^. However, the difference in age groups should be interpreted cautiously due to the larger number of patients in the older age groups than in the younger age groups. Age is an important risk factor for CVD^18^; therefore, an underlying tolerance and vulnerability to cardiovascular disease among the older age groups may explain our findings of a small decrease in the number of ED visits and the procedures observed in our study.

Many countries have introduced a variety of measures to prevent the spread of the COVID-19 virus, such as Korea, which has implemented many kinds of restrictions and rules, including social distancing measures. Even a few European countries imposed nonpharmaceutical interventions, such as lockdowns, where there was a high incidence rate of COVID-19^19^. In previous studies from China, Spain, Italy, Germany, and the United States, negative effects of national lockdowns on ED visits and emergency care in patients with cardiovascular and cerebrovascular diseases were observed, with a reduction of 40–70% in ED admissions and proper procedures for STEMI and strokes^9,10,19–21^. These results are consistent with our findings. However, in the case of Finland, even though the total number of ED visits decreased by 16% after the lockdown, the visit rate due to acute myocardial infarctions or cerebral strokes remained stable during the study period^22^. The changed government policies altered the circumstances of hospitals, and the hospitals tried to adapt their stances to these policies.

Not only were the systems of hospitals adjusted, but the patients also changed their attitudes regarding visiting the hospitals, even the EDs. Some patients who were not infected with the COVID-19 virus but had other diseases were reluctant to visit the hospitals because of the fear of transmission^7^. Outpatients in India were hesitant about regular physician visits and delayed or avoided unneeded visits due to higher caution of contracting COVID-19^23^. This phenomenon was caused by the past issue of Middle East Respiratory Syndrome (MERS) in Korea in 2015. There were intrahospital transmissions from patient to patient in EDs or hospitals and interhospital transmissions from hospital to hospital due to the transfer of confirmed patients^24,25^.

Ironically, the number of overall ED visits and executed procedures increased the most in June 2020. This was when the aggregation of confirmed cases was low, with a daily average of 39.3 cases, and social distancing was converted to the degree of daily life^2^. A previous study reported that when comparing the monthly cancer screening rates in 2019 and 2020, the figure increased in June compared to March 2020^26^. Unlike cancer screening, it is extremely difficult to delay ED visits or endure sickness without urgent action due to the characteristics of CVD itself, well known as being time-sensitive. Therefore, it is hard to interpret whether patients were merely putting off or suspending ED visits for CVDs. Rather, it would be better to interpret it as the occurrence of a sequential increase provoked by the complications of severe patients or long-COVID sequelae of the confirmed patients during the first epidemic wave. The fact that COVID-19 may contribute to symptoms related to CVDs, such as myocardial infarction or thrombosis, has been revealed^27,28^. This might further lead to the possible explanation for the observed overall increase in the treatments of CVD patients.

The southwestern region showed an exceptional increase in the number of overall ED visits and executed procedures compared to the Daegu·Gyeongbuk and central regions. In 2020, the southwestern region was affected less severely than the Daegu·Gyeongbuk region (Supplementary Table S5)^2,29^. It is possible that the collateral damage induced by measures imposed on the system of EDs (e.g., triage) and emotional avoidance related to hospital visits might be less in the southwestern region.

The current study has some limitations to be noted with caution. First, we used the customized NHID data, including information only from 2017 up to 2020. Therefore, we could not observe the longer prepandemic period before 2017 and reflect the latest trends in this study. Second, we could not conduct an analysis of ED visits according to specific types of CVDs, such as ACS, heart failure, and arrhythmia. The code ‘V192’, which we used to define CVD patients, only includes specific CVDs with high medical expenses, as shown in Supplementary Table S1. Nevertheless, the use of the code ‘V192’ helped to increase the validity of CVD diagnosis when using claim data. This specific code is not easily assigned compared to diagnosis codes, which may include the differential diagnosis; therefore, we believe that it is more likely that CVD patients who truly need acute care were detected in this analysis. Third, we could not investigate symptom-based causes of the CVD patients’ ED visits, including chest pain, dyspnea, and syncope. The NHID provides the history of ED visits but does not have information on the detailed reasons. Lastly, there was a lack of information regarding the severity of CVDs in the NHID. Instead, we observed it based on the CVD-specific deaths reported by the Korean Statistical Information Service^29^. While the overall age-standardized mortality (ASMR) decreased in 2020 compared to 2018–2019 regardless of the regions, Daegu·Gyeongbuk still exhibited the greatest increase of 11.3 per 100,000 population in the crude mortality rate and the smallest decrease of 2.4 in the ASMR (Supplementary Table S6). Although the mortality statistics were presented for the general population, it could be interpreted that the severity of CVDs remained higher in the epicenter of the COVID-19 pandemic.

However, to the best of our knowledge, our study is the first to document the impact of the COVID-19 pandemic on the emergency department visits of newly diagnosed CVD cpatients to stress the likely collateral damage caused by the pandemic in Korea. We discovered a decrease in the number of CVD patients with new ED visits and emergent procedures by month, age, and region. This result indicated that collateral damage occurred as postponed responses to acute CVDs. Additionally, it calls for the importance of preparation against an unexpected pandemic. From the public health perspective, our study provides essential evidence to arrange systems and policies to deliver proper health care services within an appropriate time during the pandemic era. Further research is needed to investigate the growing burden of mortality and the other consequences of collateral damage in patients with cardiovascular or even cerebrovascular diseases in Korea during the COVID-19 pandemic.

## Conclusion

In conclusion, the number of CVD patients with new ED visits in Korea was affected in 2020 compared to the prepandemic period. The overall number of CVD patients with new ED visits decreased, whereas the number of those with executed treatments increased. However, this trend differed by region and month, following the surge of COVID-19 clusters. The Daegu·Gyeongbuk area had the greatest reduction in the number of new ED visits and treatments in March throughout 2020. This indicates that CVD patients living in the epicenter of the COVID-19 pandemic may experience difficulty accessing health care facilities and receiving proper treatment. Future studies are required to examine the growing burden of mortality as a consequence of the COVID-19 pandemic.

### Supplementary Information

Supplementary Tables.

## Data Availability

The customized datasets used and analyzed for the study were downloaded from the National Health Insurance Service (https://nhiss.nhis.or.kr/bd/ab/bdaba021eng.do) and available during a permitted period after the approval by the NHIS for a fee.
