# Supplementary material for: Emergency department visits of newly diagnosed cardiovascular disease patients in Korea during the COVID-19 pandemic
Source: Sci Rep. 2024 Jan 3;14:397. doi: 10.1038/s41598-023-50709-w (PMC10764744; doi:10.1038/s41598-023-50709-w)
Supplement: Supplementary file 1 — Supplementary Tables. [file 41598_2023_50709_MOESM1_ESM.docx]

**Supplementary Materials**

**Supplementary Table S1.** KCD-8 codes and definitions for cardiovascular diseases and procedures from the National Health Insurance Service coverage benefit expansion policy.

**Supplementary Table S2.** The number of total cardiovascular disease patients who visited the emergency department by month from 2017 to 2020.

**Supplementary Table S3.** The number and percentage difference of new cardiovascular disease patients who visited the emergency department by month, sex, insurance type, and insurance premium.

**Supplementary Table S4.** The number and percentage difference of new cardiovascular disease patients who visited the emergency department and underwent procedures by month, sex, insurance type, and insurance premium.

**Supplementary Table S5.** Regional comparisons of the area and the population per emergency medical center and the number of newly confirmed COVID-19 cases per hundred thousand people in 2020.

**Supplementary Table S6.** The number of deaths, crude mortality rate, and age-standardized mortality rate by cause of death and region.

**Supplementary Table S1.** KCD-8 codes and definitions for cardiovascular diseases and procedures from the National Health Insurance Service coverage benefit expansion policy.

| **Code** | **Definition** |
| --- | --- |
| **Diseases** | |
| D15.1 | Benign Neoplasm of Heart |
| I01 | Rheumatic Fever with Heart Involvement |
| I05-I09 | Chronic Rheumatic Heart Diseases |
| I20-I25 | Ischemic Heart Diseases |
| I26, I28 | Pulmonary Heart Disease and Diseases of Pulmonary Circulation |
| I30-I51 | Other Forms of Heart Disease |
| I70.0 | Atherosclerosis of Aorta |
| I71 | Aortic Aneurysm and Dissection |
| I79.0, I79.1 | Disorders of Arteries, Arterioles and Capillaries in Diseases Classified elsewhere |
| M31.4 | Aortic Arch Syndrome [Takayasu] |
| Q20 | Congenital Malformations of Cardiac Chambers and Connections |
| Q21 | Congenital Malformations of Cardiac Septa |
| Q22 | Congenital Malformations of Pulmonary and Tricuspid Valves |
| Q23 | Congenital Malformations of Aortic and Mitral Valves |
| Q24 | Other Congenital Malformations of Heart |
| Q25 | Congenital Malformations of Great Arteries |
| Q26.0 | Congenital Stenosis of Vena Cava |
| Q26.1 | Persistent Left Superior Vena Cava |
| Q26.2 | Total Anomalous Pulmonary Venous Connection |
| Q26.3 | Partial Anomalous Pulmonary Venous Connection |
| Q26.4 | Anomalous Pulmonary Venous Connection, Unspecified |
| Q26.8 | Other Congenital Malformations of Great Veins |
| Q26.9 | Congenital Malformation of Great Vein, Unspecified |
| S25 | Injury of Blood Vessels of Thorax |
| S26 | Injury of Heart |
| **Procedures** | |
| OA640-OA641, OA647-OA649, O1640-O1641, O1643-O1649 | Aortocoronary Bypass Operation |
| O1660 | Repair of Cardiac Wound |
| O1671 | Closure of Patent Ductus Arteriosus ligation |
| O1672 | Closure of Patent Ductus Arteriosus division |
| O1680 | Correction of Coarctation of Aorta |
| O1690 | Closed Mitral Commissurotomy |
| O1701, O1702 | Shunt Procedure |
| O1703, O1704 | Pulmonary Artery Banding |
| O1705 | Atrial Septotomy |
| O1710, O1711 | Operation of Atrial Septal Defect |
| O1721-O1723 | Operation of Ventricular Septal Defect |
| O1730, O1740, O1750, O1760 | Operation of Valvular Stenosis |
| O1770 | Operation of Atrial Septal Defect and Pulmonary Valvular Stenosis |
| O1781-O1783 | Valvuloplasty |
| O1791-O1793, O1797 | Valve Replacement |
| O1794-O1796, O1798 | Reoperation of Valvuloplasty |
| O1799 | Sutureless Aortic Valve Replacement |
| O1800 | Total Correction of Tetralogy of Fallot |
| O1810 | Operation of Ventricular Septal Defect and Pulmonary Valvular Stenosis |
| O1821, O1822 | Repair of Endocardial Cushion Defect |
| O1823 | Left Ventricular Aneurysmectomy |
| O1824 | Left Ventricular Volume Reduction Surgery |
| O1825 | Left Ventricular Outflow Track Augmentation |
| O1826 | Right Ventricular Outflow Track Reconstruction |
| O1830 | Coronary Endarterectomy |
| O1840 | Repair of Ruptured Aneurysm of Sinus Valsalva |
| O1841 | Repair of Arteriovenous Malformation |
| O1851, O1852 | Repair of Complicated Congenital Heart Diseases |
| O1861 | Left And Right Pulmonary Artery Reconstruction |
| O1873, 1874 | Functional Correction of Single Ventricle |
| O1875 | Rastelli'S Operation |
| O1878 | Repair of Total Anomalous Pulmonary Venous Return |
| O1879 | Repair of Transposition of Great Arteries |
| O0881-O0883, O0886-O0889 | Ventricular Assist Device Treatment |
| O1890 | Extracorporeal Circulation by Heart-Lung Machine |
| O1895 | Open Cardiac Massage |
| O1901-O1904 | Partial Extracorporeal Circulation |
| O1910 | Local Perfusion |
| O1921, O1922 | Intra-aortic Balloon Pump |
| O1931 | Pericardiostomy |
| O1932, O1935 | Creation of Pericardial Window |
| O1940 | Pericardiectomy |
| O1950 | Pulmonary Artery Embolectomy |
| O1960 | Closure of Aorto-Pulmonary Window |
| O1970 | Removal of Intracardial Foreign Body |
| O1981, O1982 | Resection of Cardiac Tumor |
| O2001, O2004, O2005, O0203-O0210 | Implantation of Cardiac Pacemaker |
| O2006, O2007 | Operation of Arrhythmia |
| O0211, O0212, O2211, O2212 | Implantation of Cardioverter Defibrillator |
| O2031-O2033 | Resection of Aneurysm |
| M6510 | Percutaneous Closure of Patent Ductus Arteriosus |
| OZ751 | Percutaneous Closure of Interatrial Septal Defect |
| M6513 | Percutaneous Closure of Muscular Ventricular Septal Defect |
| M6521, M6522 | Percutaneous Atrial Septostomy |
| M6531-M6533 | Percutaneous Valvuloplasty |
| M6541-M6543, M6546-M6548 | Radiofrequency Ablation of Arrythmia |
| M0651, M0652 | Cryoablation of Arrhythmia |
| M6551-M6554 | Percutaneous Transluminal Coronary Angioplasty |
| M6561-M6564, M6565-M6567 | Percutaneous Transcatheter Placement of Intracoronary Stent |
| M6571, M6572 | Percutaneous Transluminal Coronary Atherectomy |
| M6580-M6582 | Transcatheter Aortic Valve Implantation |
| M6585 | Percutaneous Pulmonary Valve Implantation |
| M6595-M6597 | Percutaneous Transluminal Angioplasty |
| M6603-M6605 | Percutaneous Intravascular Installation of Metallic Stent |
| M6611-M6613 | Percutaneous Intravascular Installation of Stent Graft |
| M6620 | Percutaneous Intravascular Atherectomy |
| M6632, M6634, M6638, M6639 | Percutaneous Thrombus Removal |
| M6644 | Embolization |
| Q8080 | Heart Transplantation |
| Q8103 | Lung Transplantation-Heart and Lung |

**Supplementary Table S2.** The number of total cardiovascular disease patients who visited the emergency department by month from 2017 to 2020.

|  | **2017** | | **2018** | **2019** | **2020** |
| --- | --- | --- | --- | --- | --- |
| Jan | 9,885 | | 11,203 | 11,543 | 11,064 |
| Feb | 8,674 | | 8,016 | 8,321 | 8,780 |
| Mar | 9,708 | | 9,552 | 9,632 | 8,641 |
| Apr | 8,614 | | 9,527 | 9,742 | 8,506 |
| May | 8,879 | | 8,967 | 9,662 | 9,405 |
| Jun | 8,557 | | 8,295 | 8,902 | 9,787 |
| Jul | 8,825 | | 9,362 | 9,996 | 9,870 |
| Aug | 8,786 | | 8,660 | 8,969 | 8,563 |
| Sep | 8,092 | | 7,461 | 8,578 | 7,997 |
| Oct | 7,855 | | 9,045 | 9,429 | 9,454 |
| Nov | 9,453 | | 9,469 | 9,629 | 9,865 |
| Dec | 8,798 | | 9,284 | 10,176 | 9,568 |
| Sum | 106,126 | | 108,841 | 114,579 | 111,500 |
| Difference (%)^1^ | |  | 2.56 | 5.27 | -2.69 |

^1^Percentage difference of the total number of the patients who visited the emergency department in the corresponding year compared to the last one year.

**Supplementary Table S3.** The number and percentage difference of new cardiovascular disease patients who visited the emergency department by month, sex, insurance type, and insurance premium.

|  |  | Jan | Feb | Mar | Apr | May | Jun | Jul | Aug | Sep | Oct | Nov | Dec | Sum |
| --- | --- | --- | --- | --- | --- | --- | --- | --- | --- | --- | --- | --- | --- | --- |
| **Total** | | | | | | | | | | | | | | |
|  | 2018-2019 | 9,187 | 7,298 | 8,539 | 8,471 | 8,201 | 7,514 | 8,367 | 7,697 | 7,009 | 8,000 | 8,287 | 8,472 | 97,041 |
|  | 2020 | 8,601 | 7,791 | 7,334 | 7,421 | 8,135 | 8,388 | 8,553 | 7,358 | 6,865 | 8,030 | 8,441 | 8,121 | 95,038 |
|  | Difference (%) | -6.38 | 6.76 | -14.11 | -12.4 | -0.8 | 11.63 | 2.22 | -4.4 | -2.05 | 0.38 | 1.86 | -4.14 | -2.06 |
| **Sex** | | | | | | | | | | | | | | |
| Male | 2018-2019 | 6,131 | 4,922 | 5,749 | 5,617 | 5,486 | 4,988 | 5,631 | 5,149 | 4,709 | 5,392 | 5,586 | 5,687 | 65,045 |
|  | 2020 | 5,803 | 5,356 | 5,087 | 5,119 | 5,536 | 5,599 | 5,779 | 5,086 | 4,659 | 5,496 | 5,797 | 5,625 | 64,942 |
|  | Difference (%) | -5.35 | 8.83 | -11.51 | -8.87 | 0.91 | 12.25 | 2.64 | -1.22 | -1.06 | 1.94 | 3.78 | -1.09 | -0.16 |
| Female | 2018-2019 | 3,056 | 2,377 | 2,790 | 2,854 | 2,715 | 2,526 | 2,737 | 2,548 | 2,300 | 2,609 | 2,701 | 2,785 | 31,996 |
|  | 2020 | 2,798 | 2,435 | 2,247 | 2,302 | 2,599 | 2,789 | 2,774 | 2,272 | 2,206 | 2,534 | 2,644 | 2,496 | 30,096 |
|  | Difference (%) | -8.44 | 2.46 | -19.46 | -19.34 | -4.27 | 10.41 | 1.37 | -10.83 | -4.07 | -2.86 | -2.09 | -10.38 | -5.94 |
| **Insurance Type** | | | | | | | | | | | | | | |
| Self-employed  insured | 2018-2019 | 2,735 | 2,259 | 2,626 | 2,574 | 2,480 | 2,206 | 2,535 | 2,292 | 2,153 | 2,496 | 2,500 | 2,593 | 29,447 |
|  | 2020 | 2,613 | 2,466 | 2,316 | 2,268 | 2,556 | 2,600 | 2,715 | 2,303 | 2,146 | 2,449 | 2,673 | 2,540 | 29,645 |
|  | Difference (%) | -4.46 | 9.16 | -11.79 | -11.89 | 3.09 | 17.89 | 7.1 | 0.5 | -0.3 | -1.88 | 6.92 | -2.04 | 0.67 |
| Employee  insured | 2018-2019 | 5,851 | 4,560 | 5,359 | 5,329 | 5,169 | 4,813 | 5,296 | 4,905 | 4,400 | 4,951 | 5,257 | 5,351 | 61,239 |
|  | 2020 | 5,448 | 4,830 | 4,516 | 4,670 | 5,027 | 5,248 | 5,301 | 4,615 | 4,307 | 5,041 | 5,232 | 5,068 | 59,303 |
|  | Difference (%) | -6.88 | 5.93 | -15.73 | -12.36 | -2.75 | 9.04 | 0.09 | -5.9 | -2.1 | 1.82 | -0.48 | -5.29 | -3.16 |
| Medical-aid beneficiary | 2018-2019 | 602 | 480 | 554 | 569 | 553 | 496 | 536 | 501 | 457 | 553 | 530 | 528 | 6,356 |
|  | 2020 | 540 | 495 | 502 | 483 | 552 | 540 | 537 | 440 | 412 | 540 | 536 | 513 | 6,090 |
|  | Difference (%) | -10.22 | 3.23 | -9.39 | -15.04 | -0.09 | 8.98 | 0.19 | -12.18 | -9.75 | -2.35 | 1.23 | -2.84 | -4.18 |
| **Insurance Premium** | | | | | | | | | | | | | | |
| Medical aid | 2018-2019 | 602 | 480 | 554 | 569 | 553 | 496 | 536 | 501 | 457 | 553 | 530 | 528 | 6,356 |
|  | 2020 | 540 | 495 | 502 | 483 | 552 | 540 | 537 | 440 | 412 | 540 | 536 | 513 | 6,090 |
|  | Difference (%) | -10.2 | 3.2 | -9.4 | -15.0 | -0.1 | 9.0 | 0.2 | -12.2 | -9.7 | -2.4 | 1.2 | -2.8 | -4.2 |
| Bottom 25% | 2018-2019 | 1,595 | 1,272 | 1,515 | 1,493 | 1,469 | 1,300 | 1,501 | 1,343 | 1,289 | 1,425 | 1,473 | 1,510 | 17,181 |
|  | 2020 | 1,433 | 1,329 | 1,283 | 1,305 | 1,422 | 1,459 | 1,513 | 1,290 | 1,209 | 1,426 | 1,501 | 1,416 | 16,586 |
|  | Difference (%) | -10.2 | 4.5 | -15.3 | -12.6 | -3.2 | 12.3 | 0.8 | -3.9 | -6.2 | 0.1 | 1.9 | -6.2 | -3.5 |
| Bottom 50% | 2018-2019 | 1,494 | 1,194 | 1,353 | 1,341 | 1,283 | 1,198 | 1,392 | 1,282 | 1,127 | 1,328 | 1,340 | 1,335 | 15,664 |
|  | 2020 | 1,439 | 1,326 | 1,265 | 1,279 | 1,427 | 1,444 | 1,458 | 1,304 | 1,188 | 1,382 | 1,444 | 1,374 | 16,330 |
|  | Difference (%) | -3.6 | 11.1 | -6.5 | -4.6 | 11.3 | 20.5 | 4.7 | 1.8 | 5.5 | 4.1 | 7.8 | 2.9 | 4.3 |
| Top 50% | 2018-2019 | 1,997 | 1,652 | 1,925 | 1,876 | 1,779 | 1,651 | 1,846 | 1,710 | 1,559 | 1,755 | 1,826 | 1,878 | 21,450 |
|  | 2020 | 1,893 | 1,714 | 1,614 | 1,560 | 1,768 | 1,779 | 1,817 | 1,544 | 1,458 | 1,690 | 1,804 | 1,740 | 20,381 |
|  | Difference (%) | -5.2 | 3.8 | -16.2 | -16.8 | -0.6 | 7.8 | -1.5 | -9.7 | -6.4 | -3.7 | -1.2 | -7.3 | -5.0 |
| Top 25% | 2018-2019 | 3,357 | 2,613 | 3,080 | 3,078 | 2,998 | 2,761 | 2,985 | 2,760 | 2,489 | 2,825 | 3,017 | 3,098 | 35,058 |
|  | 2020 | 3,183 | 2,805 | 2,599 | 2,694 | 2,846 | 3,056 | 3,121 | 2,691 | 2,509 | 2,879 | 3,050 | 2,979 | 34,412 |
|  | Difference (%) | -5.2 | 7.3 | -15.6 | -12.5 | -5.1 | 10.7 | 4.6 | -2.5 | 0.8 | 1.9 | 1.1 | -3.8 | -1.8 |

**Supplementary Table S4.** The number and percentage difference of new cardiovascular disease patients who visited the emergency department and underwent procedures by month, sex, insurance type, and insurance premium.

|  |  | Jan | Feb | Mar | Apr | May | Jun | Jul | Aug | Sep | Oct | Nov | Dec | Sum |
| --- | --- | --- | --- | --- | --- | --- | --- | --- | --- | --- | --- | --- | --- | --- |
| **Total** | | | | | | | | | | | | | | |
|  | 2018-2019 | 6,958 | 5,536 | 6,471 | 6,603 | 6,328 | 5,787 | 6,544 | 6,018 | 5,322 | 6,193 | 6,491 | 6,447 | 74,696 |
|  | 2020 | 6,811 | 6,228 | 5,841 | 5,819 | 6,497 | 6,741 | 6,904 | 5,763 | 5,473 | 6,299 | 6,721 | 6,405 | 75,502 |
|  | Difference (%) | -2.11 | 12.50 | -9.74 | -11.87 | 2.67 | 16.50 | 5.50 | -4.23 | 2.85 | 1.71 | 3.54 | -0.65 | 1.08 |
| **Sex** | | | | | | | | | | | | | | |
| Male | 2018-2019 | 4,610 | 3,718 | 4,355 | 4,356 | 4,209 | 3,827 | 4,375 | 4,004 | 3,549 | 4,159 | 4,349 | 4,329 | 49,837 |
|  | 2020 | 4,522 | 4,268 | 4,047 | 3,995 | 4,400 | 4,492 | 4,646 | 3,943 | 3,690 | 4,305 | 4,607 | 4,449 | 51,364 |
|  | Difference (%) | -1.91 | 14.81 | -7.07 | -8.28 | 4.54 | 17.38 | 6.19 | -1.51 | 3.99 | 3.51 | 5.94 | 2.78 | 3.06 |
| Female | 2018-2019 | 2,348 | 1,819 | 2,116 | 2,248 | 2,119 | 1,960 | 2,169 | 2,014 | 1,773 | 2,034 | 2,143 | 2,119 | 24,859 |
|  | 2020 | 2,289 | 1,960 | 1,794 | 1,824 | 2,097 | 2,249 | 2,258 | 1,820 | 1,783 | 1,994 | 2,114 | 1,956 | 24,138 |
|  | Difference (%) | -2.49 | 7.78 | -15.22 | -18.84 | -1.04 | 14.77 | 4.10 | -9.63 | 0.56 | -1.97 | -1.33 | -7.67 | -2.90 |
| **Insurance Type** | | | | | | | | | | | | | | |
| Self-employed  insured | 2018-2019 | 2,066 | 1,705 | 1,984 | 1,991 | 1,918 | 1,673 | 1,959 | 1,785 | 1,619 | 1,940 | 1,938 | 1,942 | 22,517 |
|  | 2020 | 2,012 | 1,946 | 1,830 | 1,752 | 2,004 | 2,058 | 2,154 | 1,762 | 1,698 | 1,892 | 2,140 | 1,998 | 23,246 |
|  | Difference (%) | -2.61 | 14.13 | -7.74 | -11.98 | 4.51 | 23.05 | 9.95 | -1.26 | 4.88 | -2.47 | 10.42 | 2.91 | 3.24 |
| Employee  insured | 2018-2019 | 4,448 | 3,467 | 4,057 | 4,168 | 3,982 | 3,729 | 4,166 | 3,852 | 3,353 | 3,834 | 4,127 | 4,101 | 47,282 |
|  | 2020 | 4,360 | 3,889 | 3,617 | 3,692 | 4,038 | 4,259 | 4,315 | 3,636 | 3,453 | 3,988 | 4,150 | 4,002 | 47,399 |
|  | Difference (%) | -1.97 | 12.19 | -10.85 | -11.42 | 1.41 | 14.21 | 3.58 | -5.60 | 2.98 | 4.02 | 0.57 | -2.41 | 0.25 |
| Medical-aid beneficiary | 2018-2019 | 444 | 365 | 431 | 445 | 429 | 385 | 419 | 382 | 350 | 419 | 427 | 405 | 4,897 |
|  | 2020 | 439 | 393 | 394 | 375 | 455 | 424 | 435 | 365 | 322 | 419 | 431 | 405 | 4,857 |
|  | Difference (%) | -1.13 | 7.82 | -8.48 | -15.64 | 6.18 | 10.13 | 3.82 | -4.33 | -7.87 | 0.00 | 1.06 | 0.12 | -0.82 |
| **Insurance Premium** | | | | | | | | | | | | | | |
| Medical aid | 2018-2019 | 444 | 365 | 431 | 445 | 429 | 385 | 419 | 382 | 350 | 419 | 427 | 405 | 4,897 |
|  | 2020 | 439 | 393 | 394 | 375 | 455 | 424 | 435 | 365 | 322 | 419 | 431 | 405 | 4,857 |
|  | Difference (%) | -1.1 | 7.8 | -8.5 | -15.6 | 6.2 | 10.1 | 3.8 | -4.3 | -7.9 | 0.0 | 1.1 | 0.1 | -0.8 |
| Bottom 25% | 2018-2019 | 1,196 | 946 | 1,138 | 1,168 | 1,120 | 991 | 1,167 | 1,044 | 962 | 1,093 | 1,167 | 1,126 | 13,115 |
|  | 2020 | 1,109 | 1,051 | 991 | 1,006 | 1,119 | 1,162 | 1,221 | 1,000 | 946 | 1,103 | 1,162 | 1,083 | 12,953 |
|  | Difference (%) | -7.2 | 11.2 | -12.9 | -13.8 | -0.1 | 17.3 | 4.7 | -4.2 | -1.7 | 0.9 | -0.4 | -3.8 | -1.2 |
| Bottom 50% | 2018-2019 | 1,123 | 899 | 1,019 | 1,012 | 975 | 909 | 1,087 | 981 | 847 | 1,015 | 1,017 | 1,015 | 11,896 |
|  | 2020 | 1,122 | 1,036 | 1,009 | 973 | 1,116 | 1,138 | 1,161 | 1,001 | 951 | 1,066 | 1,145 | 1,083 | 12,801 |
|  | Difference (%) | 0.0 | 15.2 | -0.9 | -3.9 | 14.5 | 25.3 | 6.8 | 2.0 | 12.3 | 5.1 | 12.6 | 6.8 | 7.6 |
| Top 50% | 2018-2019 | 1,509 | 1,254 | 1,441 | 1,445 | 1,366 | 1,256 | 1,429 | 1,321 | 1,172 | 1,351 | 1,386 | 1,424 | 16,350 |
|  | 2020 | 1,468 | 1,381 | 1,287 | 1,232 | 1,384 | 1,439 | 1,449 | 1,196 | 1,148 | 1,318 | 1,425 | 1,370 | 16,097 |
|  | Difference (%) | -2.7 | 10.2 | -10.7 | -14.7 | 1.4 | 14.6 | 1.4 | -9.5 | -2.0 | -2.4 | 2.8 | -3.8 | -1.5 |
| Top 25% | 2018-2019 | 2,582 | 2,013 | 2,365 | 2,446 | 2,352 | 2,161 | 2,359 | 2,208 | 1,923 | 2,226 | 2,414 | 2,385 | 27,430 |
|  | 2020 | 2,582 | 2,274 | 2,105 | 2,147 | 2,328 | 2,486 | 2,554 | 2,132 | 2,038 | 2,305 | 2,468 | 2,392 | 27,811 |
|  | Difference (%) | 0.0 | 13.0 | -11.0 | -12.2 | -1.0 | 15.0 | 8.3 | -3.4 | 6.0 | 3.5 | 2.3 | 0.3 | 1.4 |

**Supplementary Table S5.** Regional comparisons of the area and the population per emergency medical center and the number of newly confirmed COVID-19 cases per hundred thousand people in 2020.

|  | Newly confirmed  COVID-19 cases  per 100,000 in 2020 | Area (km^2^) in 2020 | | Population (N) in 2020 | |
| --- | --- | --- | --- | --- | --- |
|  |  | Per total  emergency centers^1^ | Per regional emergency center | Per total  emergency centers^1^ | Per regional emergency center |
| Capital Region^2^ | 164.4 | 67.0 | 847.5 | 145,061 | 1,833,981 |
| Central Region^3^ | 79.9 | 418.6 | 4784.0 | 87,917 | 1,004,768 |
| Daegu·Gyeongbuk | 220.5 | 343.4 | 3983.4 | 87,000 | 1,009,195 |
| Busan·Ulsan· Gyeongnam | 67.8 | 125.0 | 2474.6 | 79,139 | 1,566,945 |
| Southwestern Region^4^ | 66.2 | 221.1 | 3252.7 | 55,917 | 822,778 |

^1^Total emergency centers include regional emergency centers, local emergency centers, and local emergency agencies.

^2^Capital (Seoul, Incheon, Gyeonggi)

^3^Central (Daejeon, Sejong, Gangwon, Chungbuk, Chungnam)

^4^Southwestern (Gwangju, Jeonbuk, Jeonnam, Jeju)

**Supplementary Table S6.** The number of deaths, crude mortality rate, and age-standardized mortality rate by cause of death and region.

|  | **CVD-specific mortality^1^** | | | | | | | | |
| --- | --- | --- | --- | --- | --- | --- | --- | --- | --- |
|  | **2018-2019** | | | **2020** | | | **Difference** | | |
|  | **N** | **Crude mortality^2^** | **ASR^3,4^** | **N** | **Crude mortality^2^** | **ASR^3,4^** | **N(%)** | **Crude mortality^2^** | **ASR^3,4^** |
| Total | 61,599 | 120.0 | 130.5 | 62,196 | 121.1 | 121.1 | 1.0 | 1.1 | -9.4 |
| Capital  Region^5^ | 24,476 | 96.0 | 122.2 | 24,371 | 94.9 | 110.9 | -0.4 | -1.1 | -11.3 |
| Central  Region^6^ | 9,361 | 133.4 | 125.5 | 9,342 | 132.8 | 115.6 | -0.2 | -0.6 | -9.9 |
| Daegu·  Gyeongbuk | 7,717 | 151.3 | 139.4 | 8,205 | 162.6 | 137.0 | 6.3 | 11.3 | -2.4 |
| Busan·Ulsan· Gyeongnam | 11,622 | 147.0 | 159.7 | 11,792 | 150.5 | 149.3 | 1.5 | 3.5 | -10.4 |
| Southwestern  Region^7^ | 8,424 | 145.1 | 122.0 | 8,486 | 147.3 | 114.5 | 0.7 | 2.3 | -7.5 |
| ^1^CVD-specific deaths defined by the ICD-10 codes ‘I00-I99’ indicating diseases of the circulatory system  ^2^Crude mortality per 100,000 population  ^3^Age-standardized mortality rate per 100,000 population  ^4^ Calculated by using mid-year population in 2020  ^5^Capital (Seoul, Incheon, Gyeonggi)  ^6^Central (Daejeon, Sejong, Gangwon, Chungbuk, Chungnam)  ^7^Southwestern (Gwangju, Jeonbuk, Jeonnam, Jeju) | | | | | | | | | |
